# Quadriceps force after TKA with femoral single radius: An in vitro study

**DOI:** 10.3109/17453674.2011.574564

**Published:** 2011-07-08

**Authors:** Sven Ostermeier, Christina Stukenborg-Colsman

**Affiliations:** Orthopaedics Department, Hannover Medical School (MHH), Germany

## Abstract

**Background and purpose:**

New implant designs have incorporated a single radius instead of a multiple radius to the femoral component in order to improve the mechanical function after TKA. We investigated the amount of quadriceps force required to extend the knee during an isokinetic extension cycle of different total knee designs, focusing on the radius of the femoral component (single vs. multiple).

**Methods:**

Human knee specimens (n = 12, median patient age 68 (63–70) years) were tested in a kinematic knee-simulating machine untreated and after implantation of 2 types of knee prosthesis systems, one with a single femoral radius design and one with a multiple femoral radius design. During the test cycle, a hydraulic cylinder, which simulated the quadriceps muscle, applied sufficient force to the quadriceps tendon to produce a constant extension moment of 31 Nm. The quadriceps extension force was measured from 120° to full knee extension.

**Results:**

The shape of the quadriceps force curve was typically sinusoidal before and after TKA, reaching a maximum value of 1,493 N at 110°. With the single femoral radius design, quadriceps force was similar to that of the normal knee: 1,509 N at 110° flexion (p = 0.4). In contrast, the multiple femoral radius design showed an increase in quadriceps extension force relative to the normal knee, with a maximum of 1,721 N at 90° flexion (p = 0.03).

**Interpretation:**

The single femoral radius design showed lower maximum extension forces than the multiple femoral radius design. In addition, with the single femoral radius design maximum quadriceps force needed to extend a constant extension force shifted to higher degrees of knee flexion, representing a more physiological quadriceps force pattern, which could have a positive effect on knee function after TKA.

Even patients with excellent results after total knee arthroplasty (TKA) have an altered walking pattern with less flexion, a shorter swing phase, and a weaker extension strength in the operated knee (Andriacchi et al. 1982, Dorr 1988, Wimmer 1999). Although patients may improve upon their preoperative extension strength by up to 50%, they do not reach the level of healthy subjects (Berman et al. 1991, Fuchs et al. 1998, 2004).

Abnormal muscle function after TKA could be due to loss of proprioreception, muscle capacity, prosthesis design, or alternations in lever arms and extension moments. With the sacrifice of the anterior cruciate ligament, the lever arm of the extensor mechanism is reduced due to a paradoxical anterior movement of the femur relative to the tibia during flexion, which results in higher quadriceps muscle forces required to extend the knee (Lewandowski et al. 1997, Dennis et al. 1998a, Ostermeier et al. 2004).

Previous biomechanical studies have shown that after stabilization of the flexion/extension axis, this paradoxical movement is reduced and the quadriceps lever arm is improved or almost restored to physiological levels, which could result in higher extension forces (Heyse et al. 2009). Hinged prostheses in particular offer this stability with improved extension force in vitro (Ostermeier et al. 2008). Non-hinged TKA designs with a single radius of the femoral condyles also offer a potential minimization of this paradoxical movement, as the flexion-extension axis is kinematically stabilized, which could increase the quadriceps lever arm (Kessler et al. 2007). Thus, the purpose of this in vitro study was to investigate the amount of quadriceps force required to extend the knee during an isokinetic extension cycle before and after total knee arthroplasty with 2 knee prosthesis systems, representing multiple and single femoral radius designs. We hypothesized that with a single femoral radius design, quadriceps force is restored to physiological levels while this is not achieved with a multiple femoral radius design.

## Methods

**Figure 1.**
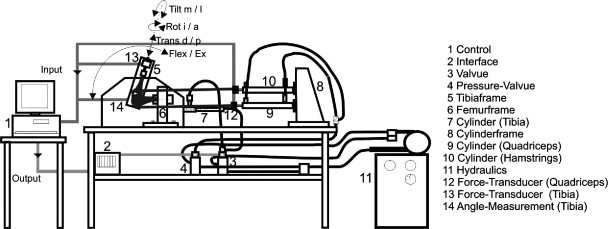
Schematic view of the test set-up according to Stukenborg-Colsman et al. (2002) and Ostermeier et al. (2008).

**Figure 2.**
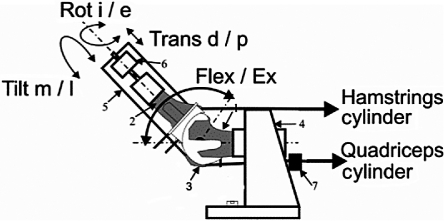
Detailed schematic side view of the applied forces and movements. The specimen was brought from a position of 120° of flexion to full extension by applying force on the quadriceps cylinder, providing a constant joint extension moment (31 Nm) resisted by the swing arm. An additional flexion force was applied by the hamstrings cylinder. 1) femur, 2) tibia, 3) patella, 4) femur frame 5) swing arm, 6) strain gauge, and 7) load cell.

The experimental in vitro setup and the test cycle we used in this study was the same as previously reported by Stukenborg-Colsman (2000, 2002) and Ostermeier et al. (2004, 2008). It simulates an isokinetic extension cycle of the knee, which allows an approximation of loadings close to the magnitude of the physiological forces and moments about the knee. 12 knee specimens of approximately the same size (median patient age 68 (63–70) years, all male) were transected 30 cm proximally and distally to the knee joint line. All surrounding tissues were preserved. The specimens were mounted into a specially designed simulator in which isokinetic flexion-extension movements were simulated (Figure 1). In this simulator, the specimens were positioned with the femur fixed horizontally and the patella facing downwards. The femoral and tibial bone stumps were fixed with bone cement in metal sleeves to reproduce the same positioning before and after removal. The tibia was attached to the simulator at mid-length by means of a linear rotational bearing, which allowed axial sliding and turning as well as rotation transverse to the axis of the tibia. The bearing itself was attached to a swing arm that allowed motion in the varus/valgus plain (Figure 2). The resulting arrangement gives complete freedom of motion of the joint, with the exception of flexion-extension, which is determined by the position of the swing-arm. The swing arm was equipped with a strain-gauge-based load-measuring device that allowed continuous monitoring of a torsional moment applied to the tibia.

The tibia was moved by the coordinated activation of 3 hydraulic cylinders, which were attached to the specimens' tendons by special clamps, one to simulate quadriceps muscle force, one to simulate a co-contraction of the hamstring muscles, and the third to apply an external flexion moment. The test cycle simulated an isokinetic extension cycle from 120° knee flexion to full extension. The quadriceps cylinder thereby applied sufficient force to the quadriceps tendon in a closed-loop control cycle to generate a constant knee extension moment of 31 Nm. The hamstrings cylinder simulated co-contraction of the hamstrings muscles with a constant co-contractive flexion force of 100 N. Initially, the swing arm was activated to bring the specimen into a position of 120° of flexion. The quadriceps cylinder was then activated in feedback control to provide a constant net joint extension moment by applying the constant extension moment at the swing arm. The joint moment was measured by the load cell in the swing arm, allowing continuous control of quadriceps force throughout the complete motion to maintain the nominal extension moment of 31 Nm. This constant extension moment was resisted by a constant swing arm flexion moment, which was generated by a third hydraulic cylinder, creating an isokinetic extension movement. After 1 complete extension cycle, the specimen could be driven back to 120° of knee flexion without remounting.

Quadriceps force was measured at a frequency of 10 Hz and with an accuracy of ± 0.1 N using a load cell (Hottinger Baldwin Messtechnik GmbH, Darmstadt, Germany) attached between the tendon clamp and the quadriceps cylinder. Degree of knee flexion was measured using a custom-made voltage goniometer attached to the tibial swing arm at a frequency of 10 Hz and with an accuracy of ± 0.05°. All test cycles were run at 20°C.

The quadriceps forces of all specimens were first measured in the normal physiological joint. The mean quadriceps force of 3 test cycles was calculated, after which a posterior cruciate retaining TKA (Triathlon; Stryker; size 5, 9-mm inlay) with no patellar resurfacing was implanted by the same surgical team in 6 of the 12 specimens, without bone cement according to the manufacturer's guidelines (i.e. “measured resection technique”). These specimens were selected randomly.

The prosthesis system offers a fixed polyethylene inlay. The tibia base-plate is implanted with a 3° posterior tibial slope. The condyles of the femoral component showed a single sagital radius in a range of tibiofemoral contact between 0 and 90°. The knee capsule and soft tissues were re-adapted and the specimen was remounted in the simulator. The test cycle was repeated as for the physiological knee.

Finally, a multiple-radius system (Interax; Stryker, Limerick; size 500, 8-mm inlay) was implanted according to the manufacturer's guidelines (i.e. “measured resection technique”) in the remaining 6 specimens. This system also provides a fixed bearing inlay and a 3° posterior tibial slope similar to the single-radius design, but the condyles of the femoral component show multiple sagital radii from 0° to 90° of knee flexion. After implantation, the specimen was remounted in the simulator and the test cycles were repeated in a similar way.

### Statistics

Since no comparison of this type of prosthesis has been quantified before, no power analysis could be done. Differences in the quadriceps force between the mean values of the experimental groups were evaluated using the non-parametric paired Wilcoxon signed-rank test at a significance level of p = 0.05, as the forces from each group were not all normally distributed. We used SPSS for Windows and Microsoft Excel for statistical analysis and randomization.

## Results

**Figure 3.**
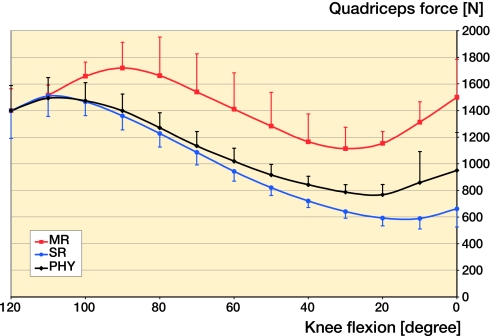
Quadriceps forces to generate an extension moment of 31 Nm under physiological knee conditions (PHY), and after implantation of a single (SR) or multiple femoral radius design (MR) from 120° of knee flexion to full extension (whiskers represent SD). Mean values of 3 repetitions.

The typical sinusoidal quadriceps force curve reached a maximum value of 1,493 N at 110° of flexion in the physiological knee (Table 1). At between 60° and 10° of knee flexion, a quadriceps force of less than 1,000 N was required to extend the knee (Figure 3). The 2 groups of the 6 knee specimens, which were randomly selected for implantation of the 2 types of knee prosthesis, showed no significant difference in maximum physiological quadriceps force (p = 0.78).

**Table 1.** Maximum quadriceps force to generate an extension moment of 31 Nm under physiological knee conditions, after implantation of a single or multiple femoral radius design

	Force **^a^**, N	SD	p-value **^b^**	p-value ^**c**^
Physiological	1,493	284		
Single-radius	1,509	209	0.4	
Multiple-radius	1,721	290	0.03	0.04

**^a^**Mean values of 3 repetitions, with standard deviation (SD).

**^b^**p-values for comparison to physiological knee conditions.

**^c ^**p-value for comparison between the 2 types of knee prostheses.

Implantation of the single-radius prosthesis (SR) resulted in slightly higher maximum quadriceps forces of 1,509 N (p = 0.4). Implantation of the multiple-radius prosthesis (MR) resulted in a higher maximum quadriceps force of 1,721 N at 90° of knee flexion (p = 0.04). Minimum quadriceps forces showed similar results, with statistically significantly higher forces needed after implantation of the multiple-radius design (Tables 2 and 3).

**Table 2.** Minimum quadriceps force to generate an extension moment of 31 Nm under physiological knee conditions, after implantation of a single or multiple femoral radius design

	Force **^a^**, N	SD	p-value **^b^**	p-value ^**c**^
Physiological	768	56		
Single-radius	589	48	0.1	
Multiple-radius	1,114	76	0.04	0.03

**^a–c^**See Table 1.

**Table 3.** Quadriceps force to generate an extension moment of 31 Nm under physiological knee conditions, after implantation of a single or multiple femoral radius design at specific knee flexion angles

	Force **^a^**, N	SD	p-value **^b^**	p-value ^**c**^
0° knee flexion				
Physiological	951	284		
Single-radius	661	135	0.03	
Multiple-radius	1,268	121	0.04	0.02
30° knee flexion				
Physiological	787	56		
Single-radius	641	50	0.04	
Multiple-radius	1,042	129	0.04	0.03
60° knee flexion				
Physiological	1,020	97		
Single-radius	944	75	0.2	
Multiple-radius	1,265	265	0.1	0.2
90° knee flexion				
Physiological	1,398	125		
Single-radius	1,360	105	0.3	
Multiple-radius	1,627	265	0.3	0.04

**^a–c^**See Table 1.

## Discussion

With this test set-up, we measured the dynamic changes in the quadriceps muscle force required to extend the knee with a constant extension moment of 31 Nm before and after TKA. A low quadriceps extension force to extend the same extension moment was considered to be biomechanically advantageous, delivering a higher degree of efficacy of the extensor mechanism. Generally, the lever arm changes during extension of the knee because of the translating tibiofemoral and patellofemoral contact points, which results in a changing quadriceps force performing a sinusoidal curve during extension (Nisell and Ekholm 1985, Ostermeier et al. 2004, 2008).

One general limitation of this in vitro test is that it only simulated one constant moment during the whole extension cycle, in contrast to the varying peak extension moments over an isokinetic extension cycle in vivo (Berman et al. 1991, Fuchs et al. 1998, 2004). Thus, the quantitative results of our study should not be translated directly to in vivo conditions. Even so, the qualitative changes we found illustrate the mechanical effect after implantation of the various knee prosthesis systems both in vitro and in vivo.

Our results differed quantitatively by about 10% from previous measurements with the same test set-up, probably because we ran the test cycles at lower temperatures. The results for the multiple-radius prosthesis type in particular were higher than the results from our previous study, as we used the fixed-inlay bearing design in the current study (Ostermeier et al. 2008). We had results similar to the findings of Andriacchi et al. (1988) and to our previous results (Ostermeier et al. 2004, 2008) with the lowest quadriceps forces under physiological knee conditions between 60° and 20° of knee flexion. Thus, the force of the quadriceps muscle is at its minimum most of the time during daily activity. Theoretically, all prosthetic systems result in altered quadriceps forces as they do not reproduce the physiological kinematics, which could be associated with a potential loss of the physiological lever arm due to insufficient restoration of the tibiofemoral and patellofemoral joint after TKA (Petersilge et al. 1994, Dennis et al. 1998a, b, Ostermeier et al. 2008). Interestingly, regarding our previous studies, this biomechanical increase in quadriceps load depended on the type of prosthesis (Ostermeier et al. 2004, 2008, Heyse et al. 2009). The more the stability of the flexion/extension axis could be restored, the lower the quadriceps force that is needed to extend the same extension moment, which is thought to increase the efficacy of the extensor mechanism. In this study, after implantation of the multiple-radius prostheses type, higher quadriceps forces were necessary to generate the same amount of extension moment compared to the conditions after implantation of the single-radius prosthesis. In addition, maximum quadriceps load following the multiple-radius prosthesis occurred at lower knee flexion angle and the forces remained higher in further extension of the knee.

Generally, following implantation of a posterior cruciate retaining prosthesis, paradoxical movement of the tibiofemoral contact point can occur due to loss of the physiological kinematic system of the cruciate ligaments, and decrease the quadriceps lever arm (Ostermeier et al. 2004, Heyse et al. 2009). In contrast, if the prosthesis design restores the physiological tibiofemoral movement, a quadriceps force curve could be produced that is similar to that under physiological conditions. As the single-radius design prosthesis showed a more stable flexion/extension axis compared to a multiple-radius design, with minimized paradoxical movement, the physiological lever arm could potentially be restored (Kessler et al. 2007). In contrast, the multiple-radius design is thought to have an insufficient reproduction of the physiological tibiofemoral contact point and lever arm, resulting in significantly higher maximum quadriceps forces needed to produce the same extension moment at lower knee flexion angles (Ostermeier et al. 2004, 2006, 2008).

Browne et al. (2005) found a significant alteration of quadriceps extension force due to the geometry of the prosthesis' patellofemoral groove or trochlea. In our study, the patellofemoral geometry of the single-radius design has a more anatomical alignment, leading to more physiological kinematics of the patella, which may be an additional reason for the reduced quadriceps forces.

Transferring these in vitro findings to the in vivo situation, prosthesis systems with a single-radius design of the femoral component condyles require adequate quadriceps muscle strength (as under physiological conditions), while the quadriceps lever arm is altered with a multiple-radius design. Thus, our findings suggest that patients with single-radius prostheses will have a mechanical advantage in knee extension compared to those with multiple-radius designs, especially regarding higher degrees of knee flexion due to the physiological quadriceps forces needed to extend the knee.
